# HIV Care in the Swedish-Danish HIV Cohort 1995-2010, Closing the Gaps

**DOI:** 10.1371/journal.pone.0072257

**Published:** 2013-08-15

**Authors:** Marie Helleberg, Amanda Häggblom, Anders Sönnerborg, Niels Obel

**Affiliations:** 1 Department of Infectious Diseases, Copenhagen University Hospital, Rigshospitalet, Copenhagen, Denmark; 2 Faculty of Health Sciences, University of Copenhagen, Copenhagen, Denmark; 3 Unit of Infectious Diseases, Department of Medicine, Huddinge, Karolinska Institutet, Solna, Sweden; 4 Department of Infectious Diseases, County Council of Gävleborg, Gävle, Sweden; 5 Division of Clinical Microbiology, Department of Laboratory Medicine, Karolinska Institutet, Solna, Sweden; Vanderbilt University, United States of America

## Abstract

**Background:**

Successful treatment reduces morbidity, mortality and transmission of HIV. We evaluated trends in the treatment status of HIV infected individuals enrolled in care in Sweden and Denmark during the years 1995-2010. Our aim was to assess the proportion of HIV-infected individuals who received services along the continuum of care in Denmark in 2010, and to discuss the findings in relation to the organization of the health care system.

**Methods:**

We analyzed CD4 counts and viral loads (VL) among all HIV patients enrolled in the cohort. For each month of the study period we estimated the proportions of patients who 1) had initiated highly active antiretroviral treatment (HAART) and had VL<500 copies/mL, 2) were not eligible for HAART, 3) had initiated HAART but had VL≥500 copies/mL, 4) were eligible for, but had not initiated HAART and 5) had initiated HAART but no VL monitoring for >13 months or 6) no HAART or monitoring of CD4 for >13 months. Patients fulfilling criteria 1 or 2 were considered successfully managed.

**Results:**

The proportion of successfully managed patients continued to increase throughout the study period and reached 83% in 2010, 92% of Swedish/Danish men who have sex with men and heterosexual patients, but only 74% of immigrants and 78% of injection drug users were successfully managed due to higher rates of inadequate monitoring in the latter two groups. In 2010, 70% of all individuals diagnosed with HIV in Denmark were virally suppressed.

**Conclusion:**

In a public health care system with free access to specialized care, successful management of the majority of HIV patients is achievable. Interventions tailored to retain immigrants and injection drug users in care are needed to further reduce the proportion of sub-optimally treated HIV patients.

## Introduction

Timely initiation of- and continuous adherence to highly active antiretroviral therapy (HAART) leads to viral suppression and immune reconstitution/preservation and thereby reduces AIDS-related and non-AIDS related morbidity and mortality [1] as well as the risk of HIV transmission [2]. In recent years a large number of efficacious and relatively non-toxic antiretroviral drugs (ARVs) has become widely available in resource-replete settings and viral suppression has become achievable in the vast majority of HIV infected individuals, even in those harboring drug-resistant virus [3]. However challenges remain: late presentation, failure to initiate therapy, poor adherence to HAART and loss to follow-up (LTFU) are obstacles for successful outcomes. A health care system with free and easy access to specialized care may be able to address these challenges successfully.

The aim of the present study was to evaluate the treatment status of HIV infected individuals enrolled in care as well as changes in the proportion, who were successfully managed in Sweden and Denmark in the period 1995-2010. Furthermore, we aimed to assess the proportion of HIV-infected individuals who received services along the continuum of care in Denmark in 2010.

## Methods

In the Swedish-Danish HIV Cohort we estimated the proportion of HIV patients who were successfully managed each month during the period of study 1995-2010. Analyses were stratified on gender, age, origin and route of HIV transmission. The continuum of care in Denmark was assessed by using data obtained from the national HIV surveillance reports and data on treatments status, viral load (VL) and CD4 counts from the Danish HIV Cohort Study.

### Setting

Denmark and Sweden have populations of 5.5 and 9.5 million, respectively, with an estimated HIV prevalence of approximately 0.08% in the adult population. Written report of all new HIV diagnoses to the national health authorities is mandatory. In Denmark HIV care is centralized in eight specialized medical centres. In Sweden there are 31 HIV care centers and 71% of patients are followed in the three largest centers, located in Stockholm (Karolinska University Hospital, South Hospital), Göteborg (Sahlgrenska University Hospital) and Malmö (Malmö University Hospital). In both countries health care and HAART are provided free of charge to all HIV-infected residents and immigrants who have or are seeking for a residence permit.

### National Guidelines for HAART Initiation

National recommendations for HAART initiation changed during the study period and differed between Denmark and Sweden. In Denmark HAART was recommended at CD4 counts ≤300 cells/µL until 2008 when this threshold was raised to 350 cells/µL. In Sweden HAART was recommended at CD4 counts ≤200 cells/µL until 2007. During 2007-2008 the threshold was 250 cells/µL and in 2009 the threshold was raised to 350 cells/µL [4]. Individuals with AIDS defining events were eligible to HAART regardless of CD4 count in both countries throughout the study period. Scheduled treatment interruptions have generally not been recommended.

### Data Sources

The Danish HIV Cohort Study, described in details elsewhere, is a population-based nationwide cohort study of all HIV-infected individuals who have been treated at Danish HIV centers after 1 January 1995 [5]. CD4 counts and VLs are extracted electronically from laboratory data files. The Swedish HIV Cohort Study was established in 2004 and collects VLs, CD4 counts as well as demographic and clinical data from an electronic clinical decision support system (InfCare HIV®), which has been implemented in all HIV care centers in the country since 2008. Data from 1995 until the implementation of the electronic system were collected in separate databases and were entered into the InfCare HIV database retrospectively. Only the three largest centers in Sweden (Karolinska, South Hospital, Sahlgrenska and Malmö) were included in the present study because data from peripheral centers were not complete before 2008.

Data on migration and vital status for Danish patients were obtained from the Danish Civil Registration System [6]. In Sweden all HIV patients who fail to attend scheduled care are tracked by the regional authorities for control and prevention of communicable diseases. Dates of death and emigration are reported to the HIV care centers where the patients were enrolled [7].

### Study Population

We included all HIV patients who were ≥18 years of age when diagnosed and treated in Denmark or at one of the three largest HIV centers in Sweden (Karolinska, South Hospital, Sahlgrenska and Malmö) between 1 January 1995 and 1 September 2010. Patients were followed from the date of HIV diagnosis, immigration or 1 January 1995, whichever came last until emigration, death or 1 September 2010, whichever came first.

### Statistics and Definitions

We assessed the treatment status of patients in the cohort once per month during the study period. If no VL or CD4 count had been measured during the month under analysis, last observation was carried forward (maximum 12 months). Patients were categorized into six groups, mentioned below: 1) had initiated HAART and had VL<500 copies/mL or initiated HAART within 6 months, 2) were not eligible to HAART according to national guidelines (specified above), 3) initiated HAART >6 months ago, and had VL≥500 copies/mL, 4) were eligible for but had not initiated HAART, 5) had initiated HAART, but no monitoring of VL for ≥13 months and 6) had not initiated HAART and no CD4 count measured for ≥13 months. Individuals fulfilling criteria 1 or 2 were considered successfully managed. Individuals in group 5 and 6 were considered inadequately monitored. As complete data on emigration and deaths were available from national registries, these events did not contribute to numbers with inadequate monitoring.

Analyses were stratified by gender, age, origin and route of HIV transmission. We constructed diagrams to visualize the proportions of patients in each of the six categories per calendar year by pooling monthly data. Furthermore, we analyzed point prevalences of successfully treated patients as of 1 July each calendar year and used logistic regression to analyze differences between groups. Analyses were adjusted for age, gender, origin (Swedish/Danish versus immigrant) and route of HIV transmission (men who have sex with men (MSM), heterosexual, injection drug users or others).

### The Continuum of Care

The continuum of care because of 1 July 2010 was assessed for Denmark only as complete nationwide data were not available for Sweden. The numbers of HIV diagnoses were obtained from the annual national HIV surveillance reports 1995-2010 [8]. The number of patients linked to care was calculated as the number of patients enrolled in the Danish HIV Cohort Study, who were diagnosed from 1995 to 2010. A patient was considered retained in care if the individual had visited an HIV care center and/or undergone measurement of VL or CD4 count within 13 months before 1 July 2010. Among patients retained in care we determined the number on HAART and the number with viral suppression (VL <500 copies/mL at the last measurement). The number of patients who died or emigrated after linkage to care and before 1 July 2010 were subtracted from the number of HIV diagnoses, and the number of patients linked to care because these patients could not be included when estimating the number and proportion of patients retained in care and virally suppressed as of 1 July 2010.

The study was approved by the Danish and Swedish Data Protection Agencies.

SPSS statistical software, Version 15.0 (Norusis; SPSS Inc., Chicago, Illinois, USA) and Stata, Version 8.0 (Stata corporation, Texas, USA), were used for data analysis.

## Results

A total of 10,136 patients were included and followed for 82,400 person-years (PY), with a median observation time of 7.8 years (interquartile range (IQR) 3.0-14.4). Characteristics of the study population are summarized in table 1.

**Table 1:** Characteristics of the study population, n (%).

Number	10,136
Total observation time (years)	82,400
Observation time (years)*	7.8 (3.0-14.4)
Deaths	1,841
Emigrations	550
*HIV cohort*	
Danish	5,519 (54.5)
Swedish	4,617 (45.6)
Male	7,497 (74.0)
Age at baseline (years)*	34 (28-42)
*Origin*	
Swedish/Danish	5,945 (58.7)
Immigrant	4,191 (41.4)
*Route of transmission*	
Men who have sex with men	4,404 (43.5)
Heterosexual	4,017 (39.6)
Injection drug use	1,156 (11.4)
Other	559 (5.5)
CD4 at baseline (cells/µL)*	334 (150-521)
*Year of diagnosis*	
≤1995	4,246 (41.9)
1996-1999	1,482 (14.6)
2000-2003	1,594 (15.7)
2004-2007	1,864 (18.4)
2008-2010	950 (9.4)

^*^median (interquartile range)

### HAART Initiation and Viral Suppression

**Figure 1:**
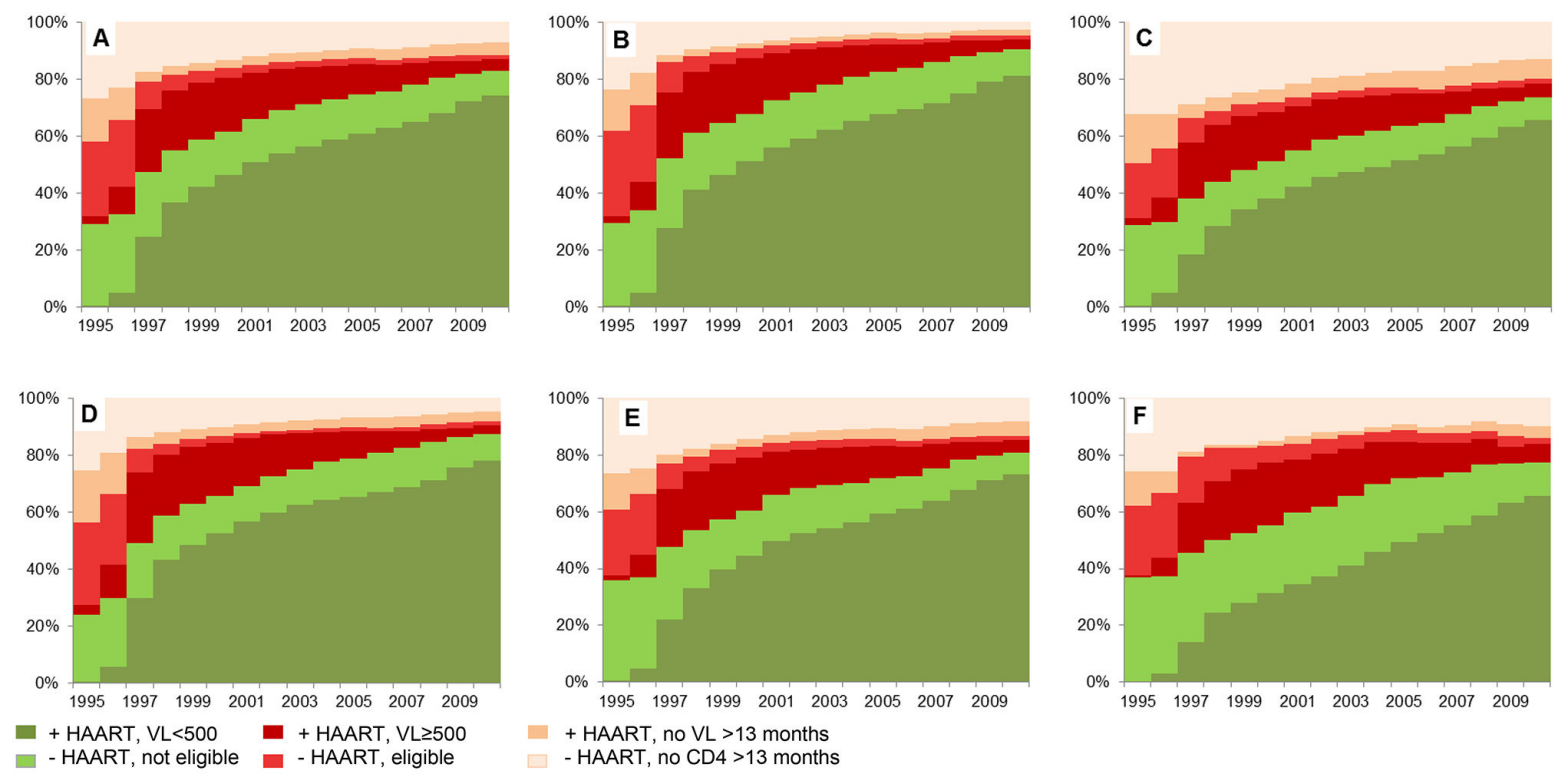
Treatment status of patients in the Swedish-Danish HIV cohort in the period 1995-2010. The panels represent all patients in the cohort (A) and stratified by origin (B: Swedish/Danish origin and C: Immigrants) and by route of HIV transmission (D: MSM, E: Heterosexuals and F: Injection drug users).

During the study period the proportion of patients on HAART increased markedly from 18.5% in 1995 to 50.1% in 1997 and 82.8% in 2010. The proportions with VL<500 copies/mL were 0.4%, 24.6% and 74.3% in 1995, 1997 and 2010, respectively. The proportion of successfully managed patients (defined above) increased from 29.2% in 1995 to 47.5% in 1997 and 83.0% in 2010 (Figure 1).

### Viraemia or Failure in Monitoring of VL after HAART

In 1995 2.6% of all patients in the cohort had VL>500 copies/mL in spite of having initiated HAART >6 months prior to VL monitoring. This proportion peaked at 21.9% in 1997 and decreased to 4.0% in 2010. Of those on HAART with monitoring of VL within 13 months, 86.2%, 47.2% and 5.2% had VL>500 copies/mL in 1995, 1997 and 2010, respectively. The proportion of patients without monitoring of VL for >13 months in spite of having initiated HAART was 15.5% in 1995, 3.6% in 1997 and 4.5% in 2010. In 1995, 1996 and 1997 53.6%, 30.8% and 3.2% of patients on HAART without monitoring of VL for >13 months had CD4 measurements within 13 months, suggesting that they were retained in care, but VL monitoring had not been implemented as routine in all centers during that period. From 1998 to 2010 this proportion was ~1%.

### Failure to Initiate HAART or in Monitoring CD4 Count

The proportion of patients who were eligible to but had not yet initiated HAART, was 26.5% in 1995, 9.9% 1997 and reached a nadir of 1.6% in 2010. The proportions who had not initiated HAART and had no CD4 measurement for >13 months were 26.3%, 17.1% and 6.9% in 1995, 1997 and 2010, respectively.

### Factors Associated with Successful Management

In 1995, young age and injection drug use or heterosexual route of transmission were associated with increased likelihood of successful management as the proportions of patients who had not yet met criteria for HAART initiation, were larger.

**Figure 2:**
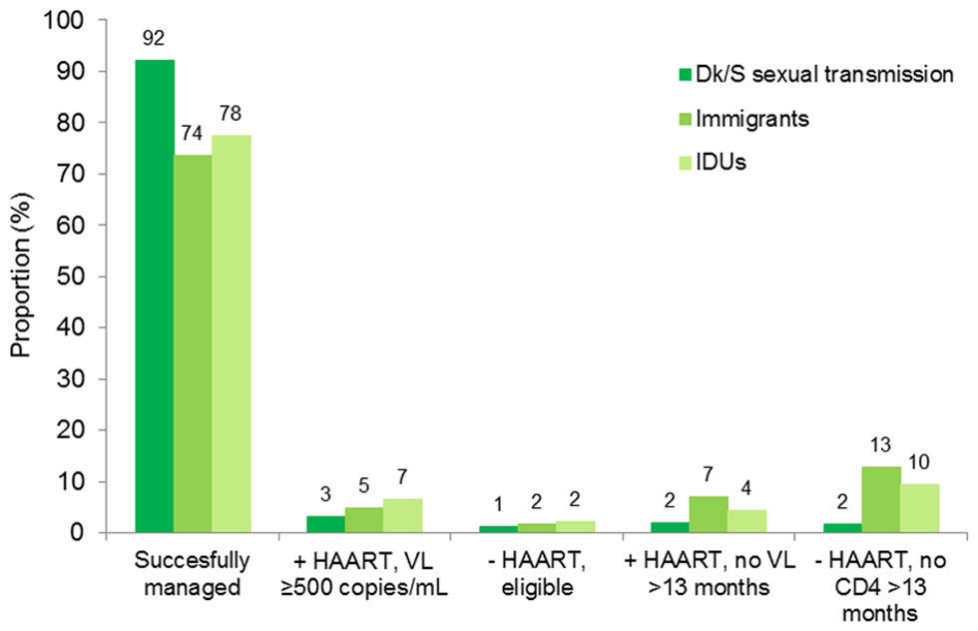
Treatment status of HIV patients in Denmark in 2010 by origin and route of HIV transmission. Successful management is defined as viral load <500 copies/mL or non-eligibility to HAART, but monitoring of CD4 within 13 months. DK: Denmark, HAART: highly active antiretroviral therapy, IDU: injection drug user, S: Sweden, VL: viral load.

After 1995, immigrants and injection drug users were less likely to be successfully managed than MSM and Swedish/Danish heterosexuals, whereas the likelihood of successful management did not differ by age (table 2). The higher proportions of unsuccessful management among immigrants and injection drug users were mainly due to inadequate monitoring which occurred more frequently prior to initiation of HAART (Figure 2). The proportion of patients with VL>500 copies/mL >6 months after HAART initiation was only 2% and 4% higher among immigrants and injection drug users, respectively, compared to Swedish/Danish MSM and heterosexuals.

**Table 2:** Proportions of successfully managed patients and odds ratios for successful management in stratified logistic regression analyses.

	1995(%)	OR (95% CI)	1997 (%)	OR (95% CI)	2010 (%)	OR (95% CI)
All	29.2		47.5		83.2	
*Age (years)*						
<35	30.7	Ref.	45.4	Ref.	80.4	Ref.
35-50	26.1	0.75 (0.64-0.88)	51.5	1.14 (0.99-1.31)	86.1	1.07 (0.9-1.3)
>50	26.2	0.59 (0.45-0.78)	50.7	1.03 (0.84-1.28)	88.5	1.12 (0.9-1.4)
*Origin/route of transmission*						
Swedish/Danish MSM	24.3	Ref.	53.3	Ref.	92.6	Ref.
Immigrant MSM	20.2	0.78 (0.60-1.01)	38.0	0.54 (0.43-0.67)	74.2	0.23 (0.19-0.29)
Swedish/Danish heterosexual male	35.0	1.72 (1.30-2.29)	55.2	1.09 (0.85-1.38)	91.0	0.81 (0.60-1.07)
Immigrant heterosexual male	28.1	1.13 (0.84-1.52)	37.3	0.53 (0.41-0.67)	70.9	0.20 (0.16-0.24)
Swedish/Danish heterosexual female	38.9	1.86 (1.38-2.51)	61.0	1.40 (1.06-1.85)	91.2	0.91 (0.64-1.30)
Immigrant heterosexual female	39.5	1.78 (1.36-2.29)	44.6	0.73 (0.58-0.92)	79.2	0.31 (0.26-0.38)
Injection drug user	34.9	1.65 (1.34-2.03)	46.2	0.74 (0.61-0.90)	78.1	0.29 (0.23-0.36)
Other/unknown	18.5	0.70 (0.48-1.01)	38.4	0.55 (0.41-0.74)	72.6	0.21 (0.16-0.28)

Odds ratios are adjusted for age, gender, origin and route of transmission.

Patients who fulfilled one of the two following criteria were defined as successfully managed: 1) had initiated HAART and viral load <500 copies/mL (monitored within 13 months) or 2) did not fulfill national criteria for initiation of HAART and CD4 count was monitored within 13 months.

CI: confidence interval, MSM: men who have sex with men, OR: odds ratio

### The Continuum of Care in Denmark

**Figure 3:**
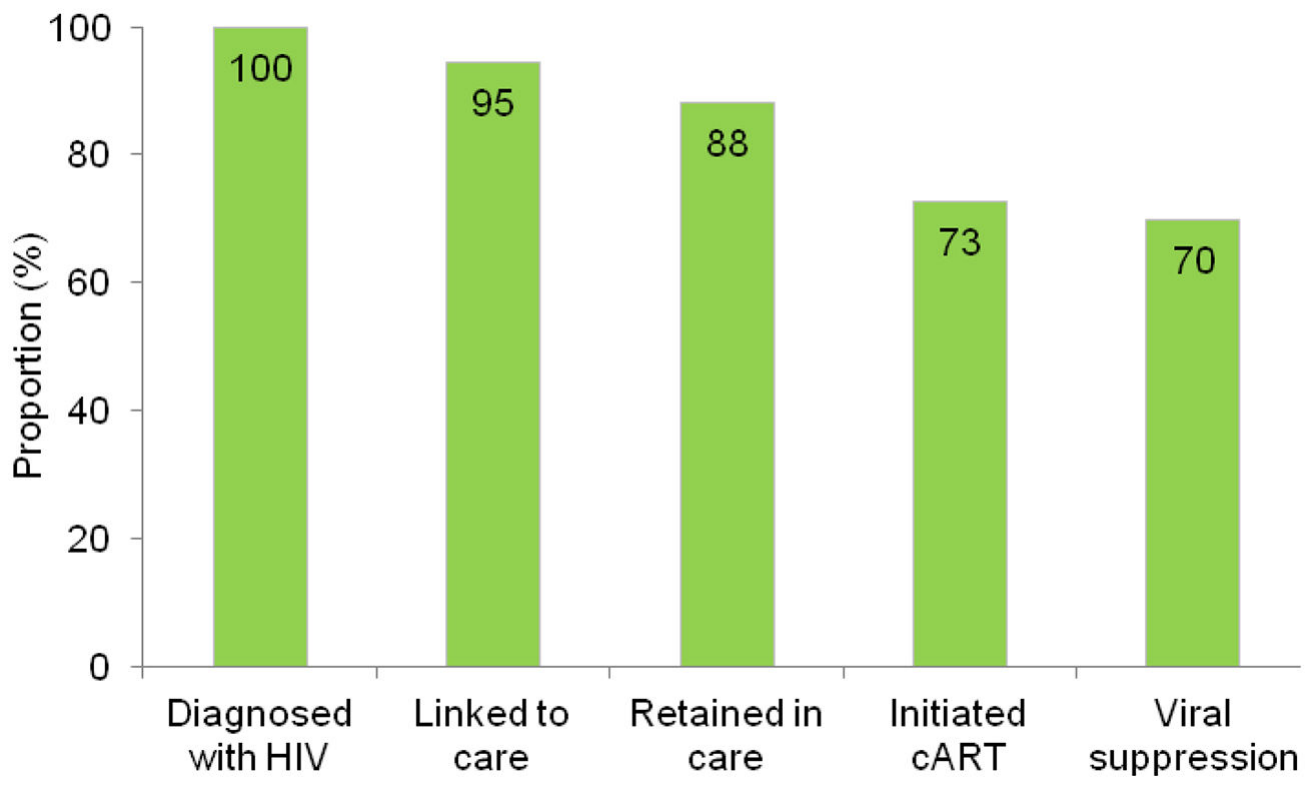
The continuum of HIV care in Denmark, 2010. Viral suppression defined as viral load <500 copies/mL.

Ninety five percent of individuals diagnosed with HIV in 1995-2010 were linked to care and 93% of those linked were retained in care (Figure 3). Among patients linked to care the median time from HIV diagnosis to first visit in an HIV care center was 14 days (IQR 2-40). Among patients retained in care, 83% had initiated HAART. Overall, 70% of individuals diagnosed with HIV were virally suppressed.

## Discussion

In this study, which includes all HIV patients enrolled in care in Denmark and the three largest HIV care centers in Sweden, we found that the proportion of successfully managed patients defined as virally suppressed or retained in care or not yet eligible for HAART, increased throughout the study period and reached 83% in 2010. Among patients of Swedish/Danish origin with sexual route of HIV transmission 92% were successfully managed, whereas this proportion was somewhat lower among immigrants and injection drug users (74% and 78%, respectively). The two latter groups were more likely to be inadequately monitored, i.e. not retained in care, especially before initiation of HAART, whereas failure to initiate HAART despite eligibility was rare.

Several factors are likely to contribute to the high proportion of successfully managed patients. In Denmark and Sweden all residents or persons applying for residence permits have access to the public health care system, which is financed through taxes [9,10]. Primary health care, investigation, specialized care, admission and treatment are free of charge. HAART is provided at HIV centers, whereas other drugs are subsidized. Health care delivery does not depend on insurance and every resident has the right to the same standard of care. Monitoring of HIV and antiretroviral therapy only takes place at hospital departments specialized in infectious diseases. In Denmark HIV care is centralized at only 8 centers, whereas only the three largest HIV care centers in Sweden were included in the study. In both countries the staff is highly trained and dedicated. To what extent the centralized and highly specialized HIV care affects treatment outcomes has not been elucidated. Programs for needle exchange and substitution therapy for substance abusers has reduced the HIV incidence among injection drug users markedly. Information campaigns and increasing knowledge and awareness of the risk of HIV may have resulted in behavioral changes and augmented this trend. Access to health care free of charge is likely to improve linkage to care. Furthermore the social security system, which ensures housing, education and day care, may also facilitate engagement in care. In spite of these measures to insure equity in access to care, we have found that outcomes were less favourable among the socially disadvantaged groups: immigrants and injection drug users. This was mainly due to inadequate monitoring – i.e. failure to retain these individuals in care prior to initiation of HAART, whereas the proportions on HAART with VL>500 copies/mL were only marginally higher among immigrants and injection drug users compared to Swedish/Danish heterosexuals and MSM. Higher rates of attrition prior to initiation of HAART are not explained by HAART being withheld or postponed for immigrants or injection drug users. Rather, regimens based on protease inhibitors are favoured if there is concern about poor adherence [11,12]. Thus the main cause of the differences in proportions managed successfully may be that the most vulnerable individuals drop out relatively soon after being linked to care. Actions such as implementation of outreach clinics/health care providers may be needed to improve outcomes in these groups [13].

The high rates of successful treatment after 1997 coincide with a substantial decrease in mortality [14]. Previous studies have shown that especially AIDS related deaths have decreased markedly [15] and that mortality among well-treated HIV infected individuals without alcohol or substance abuse or co-morbidity do not differ from that of the background population [16]. Similarly, rates of hospitalizations of HIV patients have decreased during this period [17]. Successful management ensuring that only a low proportion of patients on HAART has significant viraemia may contribute to decrease the rates of acquired HIV drug-resistance and numbers of HIV infected individuals at risk of transmitting drug-resistant virus.

We estimated that 70% of all individuals diagnosed with HIV in Denmark were virally suppressed. The Center for Disease Control and Prevention has estimated that the corresponding proportion in the United States is only 35% [18]. This difference is mainly due to lower rates of linkage to and retention in care. Each of the steps of the continuum of HIV care in the United States as well as possible effects of addressing gaps in care have been reviewed by Gardner et al. They found that incompletely engaged individuals account for the largest proportion of HIV infected individuals with detectable viremia and that multiple sequential barriers have to be overcome, since improvements in any single component of the continuum of care will have minimal impact on the proportion of HIV infected individuals with vireamia [19]. In Denmark 95% were linked to care and 93% of those linked were retained in care, whereas the corresponding numbers for the United States were 77% and 66%. In the United States socially disadvantaged groups are disproportionally affected by HIV [20], which likely partly explains the difference. But dissimilarities in the organization of the health care systems may also be important. User’s fees and lack of health insurance may be barriers to engagement in care [21]. However, differential methods for data collection and analyses may affect estimates. If nationwide data are not available or if it is not possible to link data from different registers at the individual level, the proportion of patients not retained in care may be overestimated due to migration.

In spite of the marked increase in the proportion of HIV patients with viral suppression during the study period, no decrease in incidence of HIV diagnoses has been observed. On the contrary there has been a slight increase in the annual number of newly diagnosed MSM [22,23]. This is discouraging given the hopes to the strategy of “treatment as prevention” fuelled by trials showing high efficacy of HAART in reduction of mother to child transmission and prevention of transmission in serodiscordant couples [24,2]. It has been speculated that increases in unprotected sex counterbalance the effect of reductions in community viral load [22]. An additional explanation might be that primary HIV infection drives a considerable part of HIV transmission [25] or that a large fraction of individuals living with HIV are undiagnosed [26,27]. There are no well documented estimates of numbers of individuals living with undiagnosed HIV in Denmark and whether this number has changed during the study period is unknown. Generally there has been a slight increase in the median CD4 count at time of HIV diagnosis [22] and a reduction in the proportion of patients with advanced disease at HIV diagnosis [23]. Reviews of the evidence on strategies to curtail the HIV epidemic conclude that treatment as prevention is unlikely to eliminate HIV on its own and that a combination of interventions are needed to control the epidemic [28,29].

The study has some limitations: complete data from the smaller HIV care centers in Sweden, outside the three largest cities, were not available. These centers have a relatively high proportion of individuals seeking asylum, a group for whom high rates of retention in care and adherence to HAART may be more difficult to achieve, and thus we may have overestimated the proportion of successfully managed HIV patients in Sweden. Furthermore we were not able to assess whether the outcomes of HIV care differs between large centralized centers and the smaller ones. Individuals who test HIV positive are not registered by their civil registration number in the national registry for HIV surveillance, and thus double registration cannot be completely avoided. Our calculations of the proportions of individuals diagnosed with HIV, who are linked to and retained in care and virally suppressed in Denmark, may therefore be slightly underestimated. Furthermore we were unable to assess characteristics of individuals who failed to be linked to care. When calculating the time from the first positive HIV test to the linkage to care, we largely had to rely on self-reported data from patients regarding date of first positive HIV test. We used VL<500 copies/mL as cut-off for viral suppression as this was the limit of detection at the beginning of the study period in some centers. This cut-off is higher than a commonly used definition of VL<50 copies/mL, but prevents individuals with a random “blip” from being classified as unsuccessfully treated.

Major strengths of the study are the large study population including nationwide data from Denmark and approximately 70% of HIV patients enrolled in Swedish HIV centers and the availability of valid data on vital status and migration for individuals who are not retained in care from the Danish Civil Registration System and the Swedish regional authorities for control and prevention of communicable diseases.

We conclude that in a tax-financed public health care system with easy access to specialized care free of charge, successful management of the majority of HIV patients is achievable. Interventions tailored to retain immigrants and injection drug users in care are needed to further reduce the proportion of sub-optimally managed HIV patients.
